# CuO Bionanocomposite with Enhanced Stability and Antibacterial Activity against Extended-Spectrum Beta-Lactamase Strains

**DOI:** 10.3390/ma14216336

**Published:** 2021-10-23

**Authors:** Hina Qamar, Adil Saeed, Mohammad Owais, Touseef Hussain, Kashif Hussain, Aziz ur Rahman, Sarfraz Ahmed, Sachin Kumar, Zulfiqar Ahmad Khan

**Affiliations:** 1 Interdisciplinary Biotechnology Unit, Aligarh Muslim University, Aligarh 202002, Uttar Pradesh, India; hina.dna@rediffmail.com (H.Q.); owais_lakhnawi@yahoo.com (M.O.); 2 NanoCorr, Energy & Modelling (NCEM) Research Group, Department of Design & Engineering, Bournemouth University, Poole, Dorset BH12 5BB, UK; asaeed4@bournemouth.ac.uk; 3 Department of Botany, Aligarh Muslim University, Aligarh 202002, Uttar Pradesh, India; hussaintouseef@yahoo.co.in; 4 School of Pharmacy, Glocal University, Saharanpur 247121, Uttar Pradesh, India; 2001kashif@gmail.com; 5 Department of Saidla (Pharmacy), Faculty of Unani Medicine, Aligarh Muslim University, Aligarh 202002, Uttar Pradesh, India; rahman.mdi@gmail.com; 6 Department of Microbiology, Jawaharlal Nehru Medical College, Aligarh Muslim University, Aligarh 202002, Uttar Pradesh, India; sarfrazsaifi82@gmail.com (S.A.); skpradhan90@gmail.com (S.K.)

**Keywords:** nanocomposite, copper nanoparticle, high-performance liquid chromatography, phospholipids, antibiotic resistance, extended-spectrum beta-lactamases, transmission electron microscopy, zeta potential, antibacterial activity

## Abstract

Worldwide, bacterial resistance to beta-lactam antibiotics is the greatest challenge in public health care. To overcome the issue, metal-based nanoparticles were extensively used as an alternative to traditional antibiotics. However, their unstable nature limits their use. In the present study a very simple, environmentally friendly, one-pot synthesis method that avoids the use of organic solvents has been proposed to design stable, novel nanocomposites. Formulation was done by mixing biogenic copper oxide (CuO) nanomaterial with glycerol and phospholipids isolated from egg yolk in an appropriate ratio at optimum conditions. Characterization was done using dynamic light scattering DLS, Zeta potential, high performance liquid chromatography (HPLC), and transmission electron microscopy (TEM). Further, its antibacterial activity was evaluated against the extended-spectrum beta-lactamase strains based on zone of inhibition and minimal inhibitory concentration (MIC) indices. Results from this study have demonstrated the formulation of stable nanocomposites with a zeta potential of 34.9 mV. TEM results indicated clear dispersed particles with an average of 59.3 ± 5 nm size. Furthermore, HPLC analysis of the egg yolk extract exhibits the presence of phospholipids in the sample and has significance in terms of stability. The newly formed nanocomposite has momentous antibacterial activity with MIC 62.5 μg/mL. The results suggest that it could be a good candidate for drug delivery in terms of bactericidal therapeutic applications.

## 1. Introduction

Although efficiency of antibiotics in treating several bacterial infections has been achieved, major challenges are yet to be overcome, especially bacterial susceptibility of developing resistance against multiple antibiotics. Bacteria producing extended-spectrum beta-lactamases (ESBL) and multi-drug resistance are widely available. ESBL are enzymes that enable resistance to most beta-lactam antibiotics such as penicillin, cephalosporin, and monobactam aztreonam. When resistant bacteria are exposed to β-lactam antibiotics, these enzymes are produced, thereby degrading antibiotics [1]. In the past decade, the fast expansion of multidrug resistance in certain microbial strains has resulted in the slow development of new antibiotics. Thereby, a gap between the development of new remedial measures and the appearance of antibiotic resistance in bacterial strains has become a significant health problem [2]. Several bacteria exhibit resistance to antibiotics by various means such as reducing drug degrading enzymatic activity, alterations in membrane permeability, deoxyribonucleic acid (DNA) alterations, as well as multi-drug efflux pump developments, where therapeutic drugs could not reach the target [3]. Consequently, requiring higher doses and repeated drug administration creates adverse side effects, which lead to toxicity. This complicates the metabolic process, and the potential to drug resistance increases. Recent studies have focused on solving these issues by increasing the antimicrobial efficacy of available drugs through the use of drug delivery systems that can be precisely targeted [4,5,6]. In the last few decades, to address the above problem using inorganic nanomaterials such as an antibacterial agent has looked very promising and has attracted the attention of researchers to fill the gaps where conventional antibiotics stop working [7]. Currently, the antimicrobial abilities of several nanomaterials such as silver (Ag), gold (Au), titanium (Ti), copper (Cu), palladium (Pd), etc., have been extensively explored. However, copper (Cu) and copper oxide nanomaterial address a beneficial option in contrast to the above noble metals [8,9,10]. They have gained widespread application due to their high surface-to-volume ratio, unique physiochemical properties, and cost effectiveness [8]. These nanoparticles (NP) demonstrate significant potential for multiple applications [8,9]. However, one of the major limitations that limit their use is stability. Due to their unstable nature, they have a strong tendency to aggregate and form clusters [8]. This results in the reduction of their energy related to the high surface area. Thereby, they settle down quickly, prompting the loss of antibacterial activity [9]. Current studies have been focused on the synthesis from plants by using green chemistry principles. However, even with this method achieving stability of copper nanoparticles is a major challenge, as reported in previous studies. For this reason, various coating strategies were used to obtain better biocompatibility and stability of nanoparticles in aqueous media. Among these lipids, particularly phospholipids have emerged as a versatile material [11]. Nevertheless, previous studies have reported that metallic nanoparticles can implant between lipid bilayers (liposome) or adhere to the outer surface of the liposome to form nanocomposites through delicate control of their surface chemistry [12,13,14,15]. However, this approach, even with positive results, limits their applicability as an antimicrobial agent. It is because both methods require enough nanoparticles to form a stable hybrid structure. In addition, scaling up the manufacturing process, reliability and reproducibility of the final product, use of costly chemicals (hazardous to health), and the absence of equipment and expertise limit its utilization [13].

To address all of the above issues, the present study proposes a simplified, highly versatile, and efficient method that can be employed for the design of stable nanocomposites. Previous studies have shown that CuO nanomaterial synthesized from *Momordica charantia* (MC) exhibits a thin layer of phytochemicals present in aqueous extracts that lead to agglomeration and the formation of unstable particles [16]. A novel method compiling green synthesized CuO nanomaterial from MC and phospholipids extracted from egg yolk in combination with glycerol has been presented. This method does not employ the use of organic solvents and is performed under mild circumstances. A preliminary antibacterial screening of prepared nanocomposite was performed against bacterial strains resistant to extended-spectrum beta-lactamase antibiotics to evaluate its antibacterial potential. This has been attempted for the first time to stabilize CuO nanomaterial synthesized from MC and has major potential in terms of future implementation to reproduce the method to form stable nanoparticles, which is a very challenging task for researchers [17,18]. To the best of our knowledge, it is a very simple, versatile, and highly reproducible approach over conventional strategies reported previously that requires expertise, sophisticated instruments, and toxic and costly chemicals.

## 2. Materials and Methods

### 2.1. Crude Phospholipids Isolation and Analysis

#### 2.1.1. Isolation of Crude Phospholipids from Egg Yolk

Egg phospholipids were isolated and purified following the protocol of Singleton et al., 1965, with modified variations [19]. Egg yolks from 12 eggs were separated and washed 5–6 times with acetone followed by continuous stirring in a magnetic stirrer at 1400 rpm for 5 min. White solid residues obtained after washing were collected and dried under a vacuum for 2 h to remove any traces of solvent. The resultant white powder was then processed for extraction with 1 L of absolute alcohol for 23 h with continuous stirring. Residue was re-extracted by filtering the contents. Both filtrates were mixed and evaporated at 40–45 °C. The resulting sticky mass was dried under vacuum to remove any traces of solvent. Then, the sticky mass was dissolved in 50 mL of petroleum ether, which was poured onto 100 mL of chilled acetone and was left for 2 h at a temperature of 4 °C to acquire a white sticky precipitate. Subsequently, the solvent was drawn off, and the process was repeated 2–3 times to obtain the crude form. Finally, the precipitate containing phospholipids was weighed and stored at 4 °C in chloroform to avoid any oxidation. Dried crude phospholipids isolate was dissolved in chloroform/methanol (2:1, *v*/*v*) solvents for HPLC analysis [20,21].

#### 2.1.2. HPLC Analysis

1. The HPLC System: Shimadzu Prominence Isocratic HPLC System (Kyoto, Japan), was used for the study. The system consists of an LC-20AD solvent supply unit, Rheodyne Injector, porous silica with 5 µm diameter C_18_250 × 4.6 mm column, and a UV visible SPD-20A detector system.
2. Selection of Mobile Phase: Mobile phase constituents of HPLC grade such as methanol, hexane, isopropanol, acetonitrile, and water were procured from Merck. Prior to use in the HPLC system, all solvents were filtered through a 0.22 µ membrane filter and degassed by using a sonicator. The isocratic separation of phospholipids obtained from egg yolks was accomplished with a mobile phase of acetonitrile, methanol, and 85% of phosphoric acid in a ratio of 100:10:1.8 (*v*/*v*/*v*). For the sample injection, n-hexane and 2-propanol (3:1, *v*/*v*) were used as an injecting solvent.
3. Stationary Phase: C_18_G120A column, 250 × 4.6 mm 5U with the guard column was used for the study.
4. HPLC Operating Conditions: Phospholipids were separated at room temperature. For HPLC analysis, dried crude isolate was dissolved in chloroform/methanol (2:1 *v*/*v*) solvents. The flow rate of the mobile phase was 1.5 mL/min. The detector for HPLC was UV and 204 nm wavelength.

Zeta potential was determined by using a Zetasizer that was equipped with zeta potential (Nano-ZS, Model ZEN3600).

### 2.2. Novel CuO Bionanocomposite Formulation and Their Characterization

#### 2.2.1. Preparation and Characterization of Biogenic CuO Nanomaterial

For the preparation of biogenic CuO nanomaterial our previous protocol was followed [16]. In order to synthesize CuO, a 0.1 M solution of CuSO_4_.5H_2_O was added to an aqueous extract of MC in a ratio of 1:3 (*v*/*v*) (pH 11) and was heated to 50 °C, followed by a washing and drying process. Then it was subjected to characterization by various analytical techniques as provided in the earlier study [16].

#### 2.2.2. Preparation and Characterization of Novel CuO Bionanocomposite

CuO nano-rods as synthesized above were sonicated for 5 min. To prepare CuO Bionanocomposite, phospholipids isolated from the egg yolk and cholesterol were mixed in a 2:1 (*w*/*w*) ratio and dissolved in chloroform and methanol (3:1 *v*/*v*). To 1mL of the above solution we added 3% glycerol and CuO nanomaterial (1:4 *v*/*w*), which was synthesized from *M. charantia*. The solution was then heated in a water bath at 60 °C for 15–20 min. Finally, the solution was cooled and subjected to physicochemical characterization via zeta sizer/DLS, zeta potential, and TEM.

### 2.3. Antibacterial Activity

#### 2.3.1. Antibiotic Resistance Assay

The pathogenic clinical isolates (obtained from JNMC, AMU, Aligarh) showing resistance to beta-lactam antibiotics were determined by using a standard disk diffusion method [22]. The antibiotics that were used included amoxyclav (10 µg), cefixime (10 µg), amikacin (10 µg), cefotaxime (10 µg), methicillin (10 µg), ampicillin (10 µg), oxacillin (10 µg), azithromycin (10 µg), and fusidic acid (10 µg). The assay was performed against three Gram-positive (*Bacillus cereus, Staphylococcus aureus*, and *Streptococcus mutans*) and two Gram-negative (*Escherichia coli* and *Proteus vulgaris*) clinical bacterial isolates. All bacterial strains were grown on nutrient agar plates, added impregnated antibiotic discs, and incubated at 37 °C for 24 h. The next day, the diameter of the zone of inhibition (ZoI) around the disk was measured, and the results were interpreted according to CLSI guidelines [22].

#### 2.3.2. In Vitro Antibacterial Assay

The antibacterial potential of novel formulation nanocomposite by dispersing green synthesized CuO nanomaterial in glycerol and phospholipids extracted from egg yolk against the above bacterial strains was determined by using the standard agar well diffusion method [23]. Briefly, each strain was swabbed on nutrient agar plates separately. About 50 µL of the test sample containing CuO nanomaterial, crude phospholipids, and novel nanocomposite was added to each well of a culture plate against each bacterium, respectively. Streptomycin and norfloxacin discs were used as a standard positive control. Subsequently, culture plates were incubated at 37 °C for 24 h. Antimicrobial activity was evaluated against each test organism using a zone of inhibition (ZoI) measured after the incubation period.

#### 2.3.3. Assays for Minimum Inhibitory Concentration (MIC)

It was examined using the standard broth dilution method with slight modifications [22]. To determine MIC, the first nanomaterial stock solution (1000 μg/mL) was prepared, and from this, serial two-fold dilutions (500, 250, 125, 62.5, 31.25, 15.6, 7.8, 3.9 and 0 μg/mL) were performed. Briefly, 1 mL nutrient broth and 25 µL log phase bacterial cultures were added against each dilution and incubated for 24 h at 37 °C. Bacterial growth was monitored by measuring optical density at a 600 nm wavelength using a UV–vis spectrophotometer. The MIC is defined as the lowest concentration of the antimicrobial agent that ceases 100% bacterial growth in culture.

### 2.4. Statistical Analysis

The experiment was performed in triplicate, and all data have been analyzed statistically using graph-pad instat Dataset1.ISD software (Demo version) with the ‘*t*’ test, One way ANOVA.

## 3. Results and Discussion

### 3.1. Crude Phospholipids Isolation and Characterization

**Figure 1:**
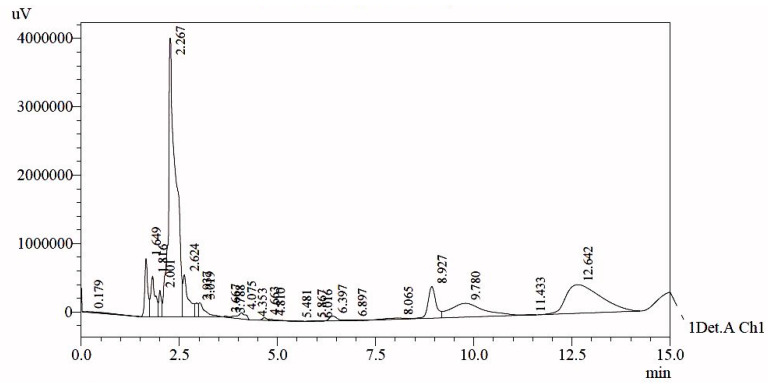
HPLC-UV chromatogram of egg yolk extract.

About 17.27g of sticky mass was obtained from the egg yolks of 12 eggs. The results showed the zeta potential of the sticky mass to be 7.74 mV. As shown in the chromatogram (Figure 1) during HPLC analysis, a total of 24 peaks were obtained and are provided in Table 1.

For further analysis of the exact composition of the peak, presented data have been compared with previously reported standard data which was collected under similar conditions on the HPLC system (Table 2) [24].

Standard phospholipids such as phosphatidylserine (PS), phosphatidylethanolamine (PE), lysophosphatidylserine (LPS), phosphatidylinositol (PI), lysophosphatidylethanolamine (LPE), phosphatidylcholine (PC), phosphatidylglycerol, lysophosphatidylcholine (LPC), and phosphatidic acid (PA) were used. When the chromatogram was obtained, it was compared with the standard data. Almost all of the peaks were obtained at the same retention time. The data showed very significant results statistically with p value equal to 0.006. Thus, nine peaks (14.2% of total phospholipids) were identified, while the remaining 15 peaks were of unknown compounds that could be further characterized in the future. These unknown peaks constituted about 85.8% of total phospholipids. As phospholipids have excellent biocompatibility and an amphibious nature, they are extensively used as drug carriers for target therapy. They are the natural building molecules of cell membranes and are identified as “pseudo-self” molecules with low allergenic potential.

### 3.2. Novel CuO Bionanocomposite Formulation and Their Characterization

#### 3.2.1. DLS and Zeta Potential Analysis of Nanocomposite Formulation

Dynamic light scattering showed an average particle size of 65 ± 5 nm with respect to intensity (percent) in function of size with a zeta potential of −7.23 mV for biogenic CuO nano-rods synthesized from *Momordica charantia*. This zeta potential indicates the unstable nature of nano-rods. In the present study, it was observed that the approximate mean particle size of biogenic CuO nanomaterial did not change when dispersed in glycerol and phospholipids at 4 °C for about 1.5 years. However, zeta potential was greatly influenced, and it increased to 34.9 mV when biogenic synthesized CuO nanomaterial was dispersed in glycerol and phospholipids were extracted from the egg yolk to form a novel formulation. This showed the particle stability of the novel nanocomposite formulation. Therefore, it can be hypothesized that the synthesized material is more stable than the previously synthesized CuO nanomaterial because of the presence of phospholipids that form a coating around the material.

#### 3.2.2. TEM Analysis of Nanocomposite Formulation

**Figure 2:**
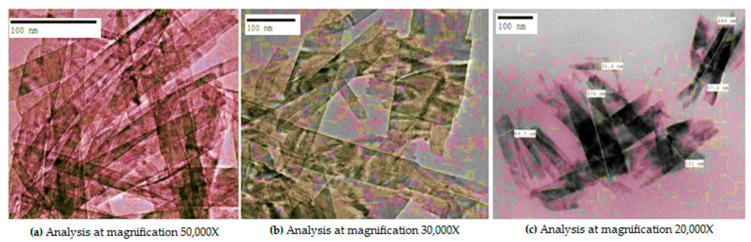
TEM analysis of CuO nano-rods synthesized from *Momordica charantia*.

Biogenic synthesized CuO nano-rods of the average particle size of 60 ± 5nm as shown in Figure 2a–c was formed. The results are in accordance with previous studies [16].

**Figure 3:**
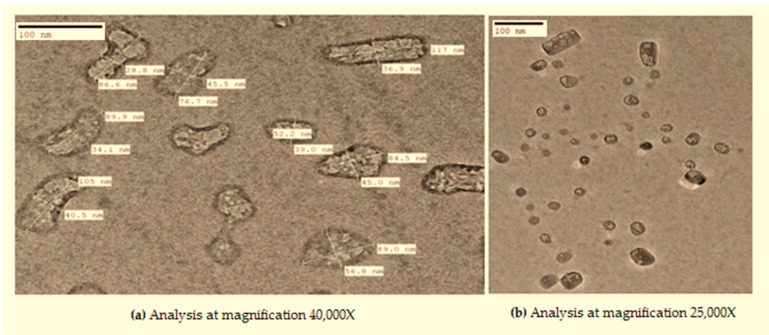
TEM analysis of novel nanocomposite (**a** and **b** representing images at different magnification).

Moreover, when these nano-rods were dispersed in phospholipids and glycerol as subjected above, a reaction proceeded. Initially, we obtained irregular shaped structures as shown below (Figure 3a) with an average particle size of 59.3 ± 5 nm. However, as the reaction progressed, these irregular distorted structures became aligned to form a proper shape (Figure 3b).

It could be hypothesized that phospholipids and glycerol react with biogenic CuO nano-rods slowly at the appropriate temperature. As the reaction proceeded with time, the unstable formulation converted to a more stable formulation. In support of our statement, we found some studies that reported the stability of nanoparticles increased when coated with phospholipids [11]. Moreover, during the synthesis process, glycerol acted as a stabilizer. Several studies have reported that glycerol is used for liposomal preparations because it enhances lipid solubility and helps in the encapsulation of materials. Glycerol interacts with the OH groups on the phospholipid chain and improves the stability of liposomes [25]. It could be hypothesized that the synthesized material would act as metal/liposome hybrids or nanocomposites. Nevertheless, there are several strategies to prepare metal/liposome nanoparticle hybrids that support this design study. Generally, the thin-film hydration method has been used, but it results in a lower yield and is deprived of selectivity of the hybrids. Removing free metallic nanoparticles that have not been encapsulated from the liposomal formulation is a tedious job and requires an additional step of separation [26,27]. Thus, the heating method of liposomal preparation involving glycerol was used. Studies reported that when liposomal constituents are heated in the presence of 3% (*v*/*v*) glycerol, hydration occurs in an aqueous medium, but to the best of our knowledge stability was not determined [25,28]. The proposed methodology is a novel approach to prepare stable structures. It is a simple, versatile, cost-effective, and eco-friendly approach everyone can use.

### 3.3. Antibacterial Activity

**Figure 4:**
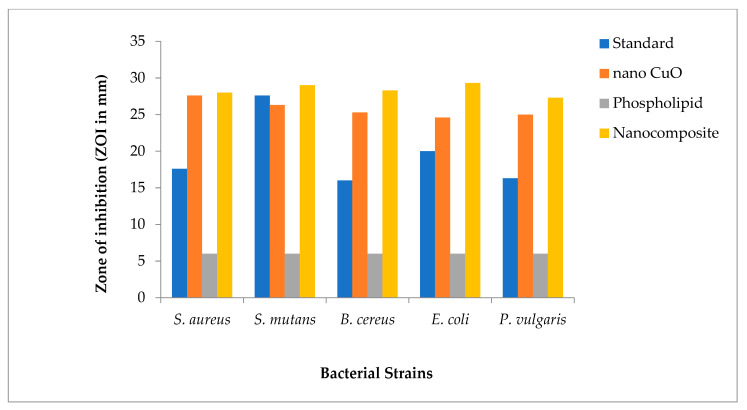
Graphical representation of comparative data for antibacterial activity of nanocomposites.

All bacterial strains tested against β-lactam antibiotics showed resistance to bacterial infections. The antibacterial activities of nanocomposites (formed by dispersing green synthesized CuO nanomaterial in glycerol and phospholipids extracted from egg yolk) against extended-spectrum β-lactamases were enhanced for both Gram-positive and Gram-negative bacterial strains when compared with CuO nanomaterial and phospholipids alone, as shown in graphical representation (Figure 4).

**Figure 5:**
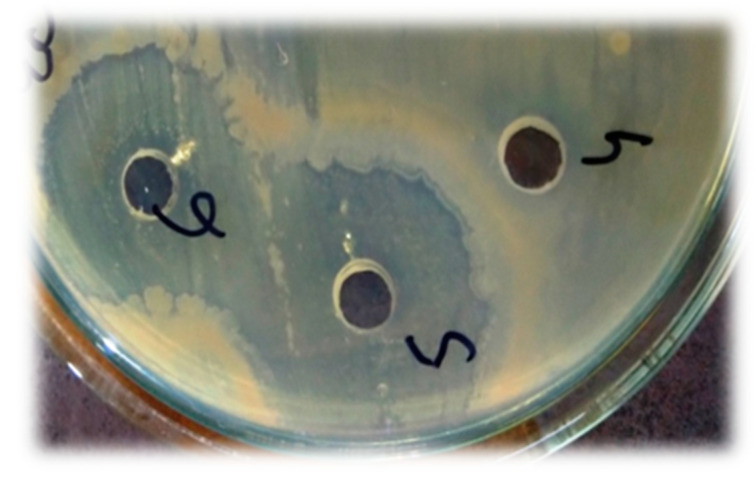
Antibacterial activity showing the zone of inhibition in *E. coli* (phospholipids showing negative activity while the nanocomposite represents the lysis zone).

The antibacterial potential of novel nanocomposites for all bacterial strains was found to be significant as compared to the standard drug with *p* < 0.0001 in most cases. The highest ZOI was obtained in the *Escherichia coli* strain (Figure 5).

The values for ZOI in different bacterial strains in decreasing order are as follows: *Escherichia coli* (ZOI = 29.3 mm) > *Streptococcus mutans* (ZOI = 29 mm) > *Bacillus cereus* (ZOI = 28.3 mm) > *Staphylococcus aureus* (ZOI = 28 mm) and *Proteus vulgaris* (ZOI = 27.3 mm) (Table 3).

The MIC for all bacterial strains was found to be 62.5 μg/mL, except *Proteus vulgaris,* which showed MIC of 125 μg/mL (Table 4). The antibacterial activity of the novel nano formulation is high; it might be because of several factors involved in the process. For example, it might be the working of phospholipids and copper oxide nanomaterial together that enhances the antibacterial activity, or phospholipids control the release of ions, thus increasing the antimicrobial potential of the nanocomposite [29,30]. It could also be proposed that phospholipids increase the surface area of copper oxide nanomaterial and, thus, antibacterial activity increases [31]. Moreover, the phospholipids stabilize the nanocomposite structure along with the steric hindrance; therefore, no particle aggregation was observed, ensuring a highly efficient surface contact area for interacting with cell surfaces [32]. In addition, the nanocomposite adheres to the cell wall, causing a leakage of intracellular proteins and other biomolecules that eventually destroy microbes [33]. Besides the above metal ion release and non-oxidative stress mechanisms, an important mechanism that ROS-induced oxidative stress generates is reactive intermediates that have strong, positive redox potential. The CuO NPs induce superoxide radical(O^−^^2^), hydroxyl radical (−OH), hydrogen peroxide (H_2_O_2_), and singlet oxygen (O_2_) cascades of reactive oxygen species (ROS) that exhibit different levels of antibacterial activity. It was observed that hydroxyl radical (−OH) and O_2_cause more microbial death than H_2_O_2_ and superoxide radicals(O^−^^2^) [34].

## 4. Conclusions

The present study concludes that the formed nanocomposite could soon find its place in nanomedicine. Studies have shown that CuO nanomaterial synthesized by green chemistry principles was unstable and aggregated to form clusters. However, its stability increased when coupled with phospholipids extracted from egg yolk and glycerol. Thus, it could be concluded that the phospholipids and glycerol act as capping agents and stabilizers. Nonetheless, design rules for particle synthesis, capping, encapsulation efficiency, hydrophobic bilayer thickness and chemistry, the influence of lipid composition on the structure of nanocomposite, etc., still have to be explored as much is yet to be investigated. Copper is eight times as economical as silver. So, more scientific efforts should be made in the preparation of copper nanocomposites. New commercial products based on copper phospholipid glycerol nanocomposites should be made as they are more stable in contrast to copper nanoparticles alone. As they have excellent antibacterial properties, they could find their place in the biomedical field to treat several bacterial infections in the near future.

## Figures and Tables

**Table 1:** HPLC data obtained from egg yolk extract.

Peak	Retention Time	Area	Height	Area %
1	0.179	727,380	10,304	0.593
2	1.649	4,608,715	850,796	3.757
3	1.816	4,712,412	593,060	3.841
4	2.001	1,812,827	385,838	1.478
5	2.267	53,024,774	4,071,016	43.222
6	2.624	5,901,699	613,187	4.811
7	2.937	1,075,792	201,297	0.877
8	3.019	2,332,563	204,673	1.901
9	3.667	100,024	16,615	0.082
10	3.788	52,192	15,557	0.043
11	4.075	1,360,125	92,329	1.109
12	4.353	4917	1220	0.004
13	4.663	329,800	37,805	0.269
14	4.810	246,766	17,628	0.201
15	5.481	14,162	1756	0.012
16	5.867	15,985	2003	0.013
17	6.016	12,122	1357	0.010
18	6.397	974,249	68,020	0.794
19	6.897	134,439	7924	0.110
20	8.065	761,702	17,250	0.621
21	8.927	6,537,964	462,860	5.329
22	9.780	11,973,239	201,907	9.760
23	11.433	83,922	4318	0.068
24	12.642	25,883,220	419,607	21.098
Total		122,680,991	8,298,327	100.000

**Table 2:** Comparison of standard phospholipids with the egg yolk extract.

Peak	Retention Time (in Minutes)		Tentative Identification
	Egg Yolk Isolated Phospholipids	Standard Phospholipids	
1.	0.179	-	Unknown
2.	1.649	-	Unknown
3.	1.816	-	Unknown
4.	2.001	-	Unknown
5.	2.267	-	Unknown
6.	2.624	-	Unknown
7.	2.937	-	Unknown
8.	3.019	3.3	Phosphatidylserine
9.	3.667	-	Unknown
10.	3.788	3.8	Phosphatidylethanolamine
11.	4.075	4.0	Lysophosphatidylserine
12.	4.353	-	Unknown
13.	4.663	4.7	Phosphatidylinositol
14.	4.810	4.9	Lysophosphatidylethanolamine
15.	5.481	-	Unknown
16.	5.867	5.8	Phosphatidylcholine
17.	6.016	-	Unknown
18.	6.397	-	Unknown
19.	6.897	6.7	Phosphatidylglycerol
20.	8.065	7.9	Lysophosphatidylcholine
21.	8.927	-	Unknown
22.	9.780	9.5	Phosphatidic acid
23.	11.433	-	Unknown
24.	12.642	-	Unknown

**Table 3:** Interpretation of the zone of inhibition results against various bacterial strains.

Name of Bacteria	Zone of Inhibition (in mm)			
	Standard	CuO Nano-Rods	Phospholipids	Nanocomposite
*Staphylococcus aureus*	17.6 ± 0.33(0.57)	27.6 ± 0.33(0.57)	6.0 ± 0.00(0)	28 ± 0.58(0.83)
*Streptococcus mutans*	27.6 ± 0.58(0.83)	26.3 ± 0.33(0.57)	6.0 ± 0.00(0)	29 ± 2.0(3.4)
*Bacillus cereus*	16 ± 0.58(0.83)	25.3 ± 0.66(1.15)	6.0 ± 0.00(0)	28.3 ± 0.66(1.15)
*Escherichia coli*	20 ± 0.33(0.57)	24.6 ± 0.33(0.57)	6.0 ± 0.00(0)	29.3 ± 0.58(0.83)
*Proteus vulgaris*	16.3 ± 0.33(0.57)	25 ± 0.33(0.57)	6.0 ± 0.00(0)	27.3 ± 0.33(0.57)

**Table 4:** Determination of minimum inhibitory concentration (MIC) for different bacterial strains.

S. No.	Name of Bacteria	MIC (μg/mL)
1.	*Staphylococcus aureus*	62.5
2.	*Streptococcus mutans*	62.5
3.	*Bacillus cereus*	62.5
4.	*Escherichia coli*	62.5
5.	*Proteus vulgaris*	125

## Data Availability

Not applicable.
